# Supplementary material for: Short email with attachment versus long email without attachment when contacting authors to request unpublished data for a systematic review: a nested randomised trial
Source: BMJ Open. 2019 Jan 30;9(1):e025273. doi: 10.1136/bmjopen-2018-025273 (PMC6359874; doi:10.1136/bmjopen-2018-025273)
Supplement: Supplementary data [file bmjopen-2018-025273supp001.pdf]

Appendix A: Short Invitation email

Dear (ENTER NAME HERE),

I am writing to invite you to join an international collaboration investigating the usefulness of adjudicating outcomes in stroke trials. We would like to include your trial (ENTER TRIAL NAME) in a systematic review. The collaboration is led by myself, Professor Philip Bath and Professor Alan Montgomery, all at the University of Nottingham.

It is currently unclear in what circumstances adjudication is required to produce unbiased estimates of effect. In this project, entitled Adjudicating Outcomes in Stroke Trials (AOST), we wish to compare and combine analyses of the primary outcome in trials using adjudicated and unadjudicated data.

We hope that you will be interested and willing to collaborate in this project. The AOST protocol is attached for further information. We look forward to hearing from you and thank you for taking the time to read this invitation.

Best wishes,

Peter Godolphin

NIHR Doctoral Research Fellow
