# Supplementary material for: Short email with attachment versus long email without attachment when contacting authors to request unpublished data for a systematic review: a nested randomised trial
Source: BMJ Open. 2019 Jan 30;9(1):e025273. doi: 10.1136/bmjopen-2018-025273 (PMC6359874; doi:10.1136/bmjopen-2018-025273)
Supplement: Supplementary data [file bmjopen-2018-025273supp002.pdf]

Appendix B: Long Invitation email

Dear (ENTER NAME HERE),

I am writing to invite you to join an international collaboration investigating the usefulness of adjudicating outcomes in stroke trials. We would like to include your trial (ENTER TRIAL NAME) in a systematic review. The collaboration is led by myself, Professor Philip Bath and Professor Alan Montgomery, all at the University of Nottingham.

As you are aware, a range of outcomes are commonly adjudicated in stroke trials. It is currently unclear in what circumstances adjudication is required to produce unbiased estimates of effect. In this project, entitled Adjudicating Outcomes in Stroke Trials (AOST), we aim to address this question by combining information about adjudication from a number of stroke trials.

We wish to compare analyses of the primary outcome in trials using adjudicated and unadjudicated data. Therefore to collaborate in this project, we would like you to provide one of two things, either:

(1) Summary results from your trial (outcome in each arm, between group measure of effect and measure of variance/confidence interval) for the primary outcome based on: (i) adjudicated data, and (ii) unadjudicated data

(2) Anonymised individual participant data for key variables from your study to allow us to carry out analysis of the primary outcome using adjudicated and unadjudicated data. For this we will need:

- Adjudicated primary outcome
- Unadjudicated primary outcome
- Treatment arm, covariates or other data used in primary analysis
- Statistical analysis plan or other details of primary analysis

Data, and any accompanying materials, can be shared in any spreadsheet format (Excel, Stata, SAS etc.) and we will re-format the data to ensure that it fits our database. Data can be shared using your preferred method, for example secure download, email or CD/memory stick.

We aim to publish the results in a high impact peer-reviewed journal. All collaborators will see the final results and manuscript for comment/interpretation, and the results will be published under the banner

of AOST with collaborators listed by name. Our group at Nottingham has considerable experience in working with individual participant data from large, international randomised trials (including projects such as BASC, DISC, NEMAS, OAST, OT, OA-COG), and of protecting the interests of the collaborators who have shared their data/results. Your data/results will not be used for any purpose other than relating to AOST.

We believe that this study will address the lack of evidence about when adjudication of outcomes may or may not be needed in stroke trials to ensure robust results. We hope that you will be interested and willing to collaborate by sharing your data or results and (should you wish to) helping in interpretation of the results and their publication. Please let me know if you would like a copy of the AOST protocol.

We look forward to hearing from you and thank you for taking the time to read this invitation.

Best wishes,

Peter Godolphin

NIHR Doctoral Research Fellow
