# Supplementary material for: Short email with attachment versus long email without attachment when contacting authors to request unpublished data for a systematic review: a nested randomised trial
Source: BMJ Open. 2019 Jan 30;9(1):e025273. doi: 10.1136/bmjopen-2018-025273 (PMC6359874; doi:10.1136/bmjopen-2018-025273)
Supplement: Supplementary data [file bmjopen-2018-025273supp003.pdf]

Appendix C:

Table A1: Sensitivity analysis. Logistic regression model for primary outcome adjusted for variables with baseline imbalance

	Response	Adjusted Odds Ratio	95% C.I	p-value
**Short message and protocol**	36/45 (80%)	0.99	(0.33, 3.03)	0.99
**Long message**	33/43 (77%)			

Adjusted for year of publication, size of trial, if the author had multiple trials included and whether the primary contact was the corresponding author. Total N included = 88. Robust standard errors were used in model fitting.
