# Supplementary material for: Short email with attachment versus long email without attachment when contacting authors to request unpublished data for a systematic review: a nested randomised trial
Source: BMJ Open. 2019 Jan 30;9(1):e025273. doi: 10.1136/bmjopen-2018-025273 (PMC6359874; doi:10.1136/bmjopen-2018-025273)
Supplement: Author's manuscript [file bmjopen-2018-025273.draft_revisions.pdf]

BMJ Open is committed to open peer review. As part of this commitment we make the peer review history of every article we publish publicly available.

When an article is published we post the peer reviewers' comments and the authors' responses online. We also post the versions of the paper that were used during peer review. These are the versions that the peer review comments apply to.

The versions of the paper that follow are the versions that were submitted during the peer review process. They are not the versions of record or the final published versions. They should not be cited or distributed as the published version of this manuscript.

BMJ Open is an open access journal and the full, final, typeset and author-corrected version of record of the manuscript is available on our site with no access controls, subscription charges or pay-per-view fees (http://bmjopen.bmj.com).

If you have any questions on BMJ Open's open peer review process please email editorial.bmjopen@bmj.com

BMJ Open

Short versus long email content when contacting authors to request unpublished data for a systematic review: a nested randomised trial

Journal:	*BMJ Open*
Manuscript ID	bmjopen-2018-025273
Article Type:	Research
Date Submitted by the Author:	13-Jul-2018
Complete List of Authors:	Godolphin, Peter; The University of Nottingham, Nottingham Clinical Trials Unit; The University of Nottingham, Stroke Trials Unit, Division of Clinical Neuroscience<br>Bath, Philip; The University of Nottingham<br>Montgomery, Alan; University of Nottingham,
Keywords:	data sharing, Stroke < NEUROLOGY, systematic review, randomised trial

SCHOLARONE™
Manuscripts

For peer review only

Short versus long email content when contacting authors to request unpublished data for a systematic review: a nested randomised trial

Peter J Godolphin[1,2], Philip M Bath[2], Alan A Montgomery[1]

Corresponding Author: Peter J Godolphin

Corresponding Author's Email: peter.godolphin@nottingham.ac.uk

Corresponding Author's Phone Number: +44 115 823 1563

Corresponding Author's Address:

Nottingham Clinical Trials Unit, Nottingham Health Science Partners, C Floor, South Block, Queen's Medical Centre, Derby Road, Nottingham, UK, NG7 2UH

Institutions:

1: Nottingham Clinical Trials Unit, University of Nottingham, Nottingham, UK

2: Stroke Trials Unit, Division of Clinical Neuroscience, University of Nottingham, Nottingham, UK

For peer review only

Abstract:

Objective: Systematic reviews often rely on the acquisition of unpublished analyses or data. We carried out a nested randomised trial comparing two different approaches for contacting authors to request additional data for a systematic review.

Participants: Participants were authors of published reports of prevention or treatment trials in stroke in which there was central adjudication of events. A primary and secondary research active author were selected as contacts for each trial.

Interventions: Authors were randomised to be sent either a short email with a protocol of the systematic review attached ("Short"), or a longer email that contained detailed information and without the protocol attached ("Long"). A maximum of two emails were sent to each author to obtain a response. The unit of analysis was trial, accounting for clustering by author.

Primary and secondary outcome measures: The primary outcome was whether a response was received from authors. Secondary outcomes included time to response, number of reminders needed before a response was received and whether authors agreed to collaborate.

Results: 88 trials with 76 primary authors were identified in the systematic review, and of these, 36 authors were randomised to Short [trials=45], and 40 to Long [trials=43]. Responses were received for 69 trials. There was no evidence of a difference in response rate between trial arms (Short vs Long, odds ratio 1.10, 95% C.I: [0.36, 3.33]). There was no evidence of a difference in time to response between trial arms (Short vs Long, hazard ratio 0.91, 95% C.I: [0.55, 1.51]). In total, 27% of authors responded within a day and 22% of authors never responded.

Conclusions: Email length did not have an impact on the number of responses received when acquiring data for a systematic review involving stroke trials, or the time taken to receive these responses.

Keywords: data sharing, randomised trial, stroke, systematic review

For peer review only

For peer review only - http://bmjopen.bmj.com/site/about/guidelines.xhtml

Article summary:

Strength and limitations of this study:

- This is the first randomised trial comparing different email formats when contacting authors to request unpublished data for a systematic review.
- This study follows a clear and rigorous protocol, guided by experienced methodologists, and implemented in a clinical trials unit.
- Blinding was not possible, as it was clear which format of email had been sent. However contacted authors were not aware of this trial during the study and therefore we expected that their response rate and time to response would remain unbiased.
- The sample size is constrained by the number of eligible studies identified for the systematic review. It is possible that a larger study would detect small but important effects of email format on response rate.

For peer review only

For peer review only - http://bmjopen.bmj.com/site/about/guidelines.xhtml

Introduction:

Systematic reviews are often considered as the highest level of evidence available[1]. However, meta-analyses, the statistical component of a systematic review, often rely on the acquisition of unpublished summary results or further data. It can be a challenge to obtain this data, especially if the meta-analysis requires individual patient data[2], resulting in insufficient studies to pool together[3].

There are many reasons why there may be difficulties in acquiring data; authors may not wish to share their data[4], they may be too busy to respond or deal with requests[3], they may have moved institution and be uncontactable, the data could have been lost or destroyed, or authors may not have access to the data[5]. Author inclination to share data could be related to the weight of evidence and the perceived quality of statistical results from their study[6]. Research studies that report borderline evidence of an intervention effect may be less likely to take part in data sharing, which would result in publication bias for a systematic review[7]. Various methods are currently employed to encourage authors to share data for a systematic review, and a randomised trial investigating if financial incentives may be effective is currently underway[8].

There appears to be no consensus about the best way to approach authors to contact them for data. The corresponding author should be the most appropriate person to contact when attempting to acquire unpublished clinical trial data, but these authors are often in senior positions and may have little time to deal with requests. Therefore, if email contact is used, this must be clear, concise and easy to respond to. Some authors may prefer a shorter email, with additional information attached, whilst others may prefer a longer email with sufficient information they need to make a decision without reading additional attached information such as a study protocol.

The initial interaction with any author may have a large impact on whether they collaborate, or even on whether they reply at all. The first step when attempting to acquire data is to open up a channel of communication with the data custodian, assumed in this paper to be the author of the journal article. Thus, an important outcome to assess is whether an initial response is received, before considering if interaction with the author affects willingness to share data.

For peer review only - http://bmjopen.bmj.com/site/about/guidelines.xhtml

We carried out a nested randomised trial, which investigated two different approaches to contacting authors for data in a systematic review, with the primary aim of seeing which method elicits the most complete response.

Methods:

Participants:

The systematic review chosen for this study investigates whether central adjudication of the primary outcome in stroke trials has any impact on the main trial primary analysis[9]. Since the information required for the review is not commonly published in the main or secondary trial publications, author contact is essential which makes it a useful setting to test the effect of different ways of contacting authors on response rate.

In brief, the studies included were those adjudged to be randomised trials of prevention or treatment of stroke, that had centrally adjudicated their primary outcome. MEDLINE, Embase, the Cochrane Central Register of Controlled Trials, Web of Science, PsycINFO and Google Scholar were searched for relevant articles. We restricted searches from Google Scholar to the first 300 articles[10], and only articles written in English were considered.

Two author contacts were chosen from each identified trial and these were the participants selected for this study. The primary contact was ideally one of the corresponding authors. This author was checked on PubMed to identify whether they were still an active researcher (paper published within the last 2 years). If the corresponding author appeared to have ceased research activity, then a secondary author who was still an active researcher (preferably first/second/last) was selected as the primary point of contact. Additionally, a second research active author was chosen as a second contact for the trial. Ideally, this second contact also had a major role in the trial, determined by their position on the main trial publication and in the author contribution section of the manuscript, if completed.

Authors were asked if they were willing to collaborate in our research programme investigating adjudication in stroke trials. In order to collaborate, they were asked to provide data in one of two formats: individual participant data or summary results.

For peer review only - http://bmjopen.bmj.com/site/about/guidelines.xhtml

Patient and Public Involvement:

Patients were not involved in this study.

Trial Design:

This study was a parallel group, randomised trial of short email and attachment versus long email to elicit response from potential collaborators. The trial was nested within a prospective meta-analysis. Blinding was not possible, as it was clear to contacted authors and the trial team which format of email had been sent. However, contacted authors were unaware that alternative formats of contact email were being compared in a nested randomised study and therefore we expected that their willingness to respond would be unbiased.

Randomisation:

Randomisation occurred at author level. This was to ensure that if any author had published multiple trials that were to be included, then they only received one email, rather than multiple emails, which could be in different formats. Randomisation was stratified by year of publication (median split: <2011/≥2011), size of trial (median split: <1738/≥1738) and if the author had multiple trials which are included (yes/no). Therefore, there were eight strata. If authors had multiple trials, then the within-author median size and median year were used when stratifying. Allocation was concealed and a researcher not involved in the trial randomised participants using a computer-generated random number list.

Participants were randomised in a 1:1 ratio to either of the two treatment groups, Short, where they received a short email message that fits on one screen without the need to scroll, with the protocol attached, or Long, where they received a longer email message that contained a fuller description of the systematic review and request for information, with the protocol available on request (see *Appendix B & C*). The protocol detailed the purpose of the systematic review, the data that was requested and the planned programme of research.

Plan for eliciting response:

Each author received a maximum of two emails until a response was received. The primary contact was first sent an email in the format of the randomly allocated intervention. If no response was received, then a second email was sent 28 days later, which included some additional brief reminder

For peer review only - http://bmjopen.bmj.com/site/about/guidelines.xhtml

text, which was the same for both groups, added at the top of the original email (achieved using Reply to all in Outlook).

If there was still no response from the primary contact author after a further three-week period, then the second contact author was sent an email in an identical format, waiting four weeks before a reminder was sent. The second contact was not emailed if the first contact responded within the seven-week timeframe. Therefore, there was a maximum of four emails in total that authors received if there was no response. Participants were followed up for a further eleven weeks after the fourth and final email. After this stage, any authors that had not responded were recorded as non-respondents, regardless of contact thereafter (Figure 1).

Outcome Measures:

The primary outcome for this nested randomised trial was whether a response was received from either of the two contacted authors in the trial timeframe. This was a binary outcome (Response received, Yes/No). Out of office automatic replies were not counted as a response, and no additional emails were sent upon receipt of an out of office reply.

Secondary outcomes were time to response, measured in days, and the number of emails needed to respond. Upon receipt of an initial response, this was categorised as either being Negative, Neutral or Positive. Positive responses were allocated if there was an interest in the collaboration and research questions, above simply asking for further information. Negative responses were assigned if participants gave the indication that they did not want to be involved/did not have time to take part. All other responses were given Neutral.

The eventual outcome upon the end of the trial timeframe was allocated as either agree to collaborate, do not agree to collaborate, no decision reached or no response. If there was an agreement in principle regardless of whether data had been received, then this was assigned as agree. If there was a clear indication that the author could not/did not want to help then this was allocated do not agree. No decision reached was given if it was still unclear whether the author would be willing to take part in the collaboration.

Statistical Analysis:

For peer review only - http://bmjopen.bmj.com/site/about/guidelines.xhtml

The sample size for this nested randomised trial was the number of studies identified in the systematic review, and although allocation was by author, the unit of analysis is the individual trial. The characteristics of each trial were summarised using appropriate descriptive statistics for each intervention arm. The primary approach to between-group comparative analyses was by intention-to-treat. The evaluation of response rate was performed using adjusted logistic regression models. The adjusted model included stratification factors; year of publication, patients randomised and multiple trials. The primary efficacy parameter comparing interventions was the odds ratio along with the corresponding 95% confidence interval. Robust standard errors were used to account for correlation between multiple trials for the same author. A sensitivity analysis was carried out by further adjusting the primary analysis model for any characteristics of trials with marked imbalance between intervention groups.

Time to response was investigated using survival analysis, with response being the outcome of interest. Adjusted hazard ratios, adjusted for stratification factors, were reported alongside 95% confidence intervals. Additional secondary outcomes were summarised using mean, standard deviation, median, lower and upper quartiles, or frequency counts and percentages where appropriate. All analyses were performed in Stata version 15.0 or later.

Results:

The study commenced on 11th July 2017. 88 trials were identified from a systematic search of the literature and were to be included in the review. This corresponded to 76 unique authors who were selected as the primary contact for these studies. 36 authors were randomised to Short (45 trials), and 40 to Long (43 trials) email formats. The majority of the studies had their main publication published between 2006 and 2015, were either primary or secondary prevention stroke trials, were carried out in over 50 trial centres and had a median number of patients randomised above 1000 (Table 1). Trials randomised to either group had similar study design, intervention type and comparator, but studies allocated to Long tended to have more participants included and a larger number of trial centres. The number of intervention groups was similar for studies allocated to either Short or Long.

For peer review only - http://bmjopen.bmj.com/site/about/guidelines.xhtml

The majority of authors chosen as the primary contact were the first author of the main publication, or listed first in the writing committee if the article was published under a collaborative group. Conversely, the last author was the most common choice for the second contact, although there was greater variation in choice for this contact, with a position other than first, second or last also prevalent. For studies allocated to Long, the primary contact was more likely to be the corresponding author compared to trials allocated to Short, although author position was similar for both groups (Table 1).

In all, responses were received for 69 trials, with authors of 19 studies (22%) never responding. There was no evidence of a difference in response rate between trial arms (Table 2 and *Appendix A*). Average time to response was marginally quicker among those allocated to Short, with a greater proportion of authors in this trial arm responding to the first email, but there was insufficient evidence to determine a difference between trial arms on time to response (Table 3 & Figure 2). In total, 27% of authors responded within a day and 38% replied within a week.

A short email elicited a more favourable first response, with few negative replies. However, this did not lead to a larger number of authors agreeing to collaborate, with many authors not following up on their positive intent. Conversely, those allocated the longer email were more likely to respond in a negative fashion, but this meant that the decision not to collaborate was established sooner, and there was a smaller proportion with no decision reached (Table 4).

Discussion:

This nested randomised trial found that email format did not have an impact on author response when attempting to acquire data for a systematic review. Authors responded in a similar timeframe, and needed a similar number of reminders before a response was received. There was some indication that authors that received a shorter email were more likely to respond positively at first, but email length did not change the likelihood of collaboration.

Approximately three-quarters of authors in the study responding regardless of email format. A similar study which attempted to contact authors to obtain data for a diagnostic accuracy review for hepatic fibrosis found a similar response rate, with 68% of authors responding to requests for data[11]. For both email types we included text which invited authors who provided data into a collaboration to

For peer review only - http://bmjopen.bmj.com/site/about/guidelines.xhtml

assist (at their discretion) with interpretation and write-up of results, and co-authorship as part of a collaborative group. Our view was that offering greater opportunity for collaboration to authors rather than simply providing data or further results might produce a greater and more complete response. A qualitative interview study which investigated strategies to access unpublished clinical trial data mentioned a lack of collaboration as a main barrier to data sharing[12]. Thus, offering the opportunity for authors to collaborate and being upfront about giving collaborators the chance to input in both interpreting results and the write-up of any papers could give a more positive and thorough response.

Whilst short emails were responded to marginally quicker on average, there was insufficient evidence to conclude benefit of this style on time to response. Selph et al.[11] found that responses were received from 23% of authors after one request, with 55% responding after just two requests (one reminder). This is similar to our study, where we found that 41% of authors had responded after one email and two emails (one reminder) elicited 62% response rate. A third email provided 70% response rate, with little further improvement following a fourth email. The number of emails sent and the number sent to each contact (if there are multiple like in our study) are additional areas where similar research could help to introduce a more rigorous evidence base.

There was some indication that authors allocated to a short email were more likely to respond positively, with only two negative first responses received from these authors, and double the number of positive responses. However, the number of authors eventually agreeing to collaborate was similar for both groups. This suggests that whilst a long email resulted in more negative initial responses, authors who did respond were aware of the implications of our study and could make an informed decision. Thus, the majority of positive first responses to those allocated to a long email turned into collaboration. It could be hypothesised that whilst those allocated to the short email had a detailed protocol attached, authors may not have opened attachments and therefore were agreeing to be involved in the study without fully realised the commitment. This led to a far larger amount of time and resource dealing with these authors, who eventually declined to collaborate, or no decision was reached in the trial timeframe.

A limitation of this study was the constrained sample size, enforced by the systematic review that the study was based on. Thus, power to detect a difference in response rate was low. However, this study does provide a framework to carry out a larger trial of this kind. A further limitation of our trial was that

For peer review only - http://bmjopen.bmj.com/site/about/guidelines.xhtml

blinding was not possible, because the investigator was responsible for sending all emails and collating the data. It was impossible to mask which intervention participants were assigned to, due to the email thread being used to reply to messages and send reminders. Therefore, there is a possibility that some outcomes are at a risk of bias. Furthermore, the eventual outcome was not expected to be greatly influenced by the intervention, and was more reliant on subsequent communication such as emails or phone contact. However, the protocol was finalised in advance of randomisation, and the trial team followed this protocol, therefore the risk of bias from the trial team was low. In addition, study authors, although aware of email format they received, were not aware of the alternative email format nor were they aware of the nested randomised study, therefore the risk of bias from participants was low.

It could be argued that this study did not investigate what could be perceived as the key question, whether data was shared. This was due to our belief that whether authors provided data was dependent on other factors, and not the original email length and style. Our study investigated an intervention which allows researchers to open up a channel of communication with the data custodian, and there is the potential to use a different intervention to evaluate how best to acquire data (for example financial incentives[8]).

This nested randomised trial showed that email length had no impact on author response, or agreement to collaborate, when attempting to acquire data in our systematic review. Further studies could test similar hypotheses to evaluate this further, with a greater sample size and in a different clinical area. Given this issue involves countless researchers who strive to generate high quality research through systematic reviews, this problem seems one that is not only important to tackle, but could be potentially simple to answer. Whilst the first question has to be whether an author responds, the more important question to answer is whether or not an author agrees to collaborate, and arguably even more important, whether or not data is shared. These questions go far beyond the scope of this small trial, but future studies building on this could investigate these issues and attempt to find an intervention that not only boosts response, but also boosts collaboration.

For peer review only - http://bmjopen.bmj.com/site/about/guidelines.xhtml

Contributors: All authors conceived the study. PJG wrote the analysis plan. AAM reviewed the analysis plan. PJG collected the data, carried out interventions and undertook data analysis. PJG, AAM, PMB interpreted the data. PJG wrote the first draft of the manuscript. AAM, PMB commented on successive versions of the manuscript for important intellectual content. All authors approved the final version of the manuscript.

Funding: PJG was funded for this summary of independent research by the National Institute for Health Research (NIHR)'s Doctoral Research Fellowship Programme (DRF-2016-09-057). The views expressed are those of the authors and not necessarily those of the NHS, the NIHR or the Department of Health. PMB is Stroke Association Professor of Stroke Medicine and is a NIHR Senior Investigator.

Competing interests: None

Ethical approval: As this was a randomised trial of a methodology rather than a treatment involving patients, we considered that prior consent and trial registration were not appropriate. Furthermore, the first contact with participants had to be randomised otherwise this would negate the purpose of this study.

Data sharing: The datasets generated during this study will be available upon request from Peter Godolphin (peter.godolphin@nottingham.ac.uk), a minimum of 6 months after publication. Access to the data will be subject to a review of a data sharing and use request by a committee, and will only be granted upon receipt of a data sharing and use agreement. Any data shared will be anonymised which may affect the reproducibility of published analysis.

References

1. Paul, M. and L. Leibovici, *Systematic review or meta-analysis? Their place in the evidence hierarchy.* Clinical Microbiology and Infection, 2014. **20**(2): p. 97-100.
2. Cooper, H. and E.A. Patall, *The relative benefits of meta-analysis conducted with individual participant data versus aggregated data.* Psychol Methods, 2009. **14**(2): p. 165-76.
3. Jaspers, G.J. and P.L. Degraeuwe, *A failed attempt to conduct an individual patient data meta-analysis.* Syst Rev, 2014. **3**: p. 97.
4. Savage, C.J. and A.J. Vickers, *Empirical Study of Data Sharing by Authors Publishing in PLoS Journals.* PLOS ONE, 2009. **4**(9): p. e7078.
5. Lundh, A., L.T. Krogsbøll, and P.C. Gøtzsche, *Access to data in industry-sponsored trials.* The Lancet. **378**(9808): p. 1995-1996.

For peer review only - http://bmjopen.bmj.com/site/about/guidelines.xhtml

6. Wicherts, J.M., M. Bakker, and D. Molenaar, *Willingness to Share Research Data Is Related to the Strength of the Evidence and the Quality of Reporting of Statistical Results.* PLOS ONE, 2011. **6**(11): p. e26828.
7. Ahmed, I., A.J. Sutton, and R.D. Riley, *Assessment of publication bias, selection bias, and unavailable data in meta-analyses using individual participant data: a database survey.* BMJ, 2012. **344**.
8. Veroniki, A.A., et al., *Contacting authors to retrieve individual patient data: study protocol for a randomized controlled trial.* Trials, 2016. **17**(1): p. 138.
9. Godolphin, P., A. Montgomery, and P. Bath. *A comparison of independent adjudication and site assessment of outcomes and its impact on treatment effect estimates in randomised stroke trials: systematic review and meta-analyses*. 2017 [cited 2018 22/03/2018]; Available from: http://www.crd.york.ac.uk/PROSPERO/display_record.php?ID=CRD42017056731.
10. Haddaway, N.R., et al., *The Role of Google Scholar in Evidence Reviews and Its Applicability to Grey Literature Searching.* PLOS ONE, 2015. **10**(9): p. e0138237.
11. Selph, S.S., A.D. Ginsburg, and R. Chou, *Impact of contacting study authors to obtain additional data for systematic reviews: diagnostic accuracy studies for hepatic fibrosis.* Systematic Reviews, 2014. **3**: p. 107-107.
12. Wolfe, N., P.C. Gøtzsche, and L. Bero, *Strategies for obtaining unpublished drug trial data: a qualitative interview study.* Systematic reviews, 2013. **2**(1): p. 31.

For peer review only

For peer review only - http://bmjopen.bmj.com/site/about/guidelines.xhtml

Tables and Figures:

Table 1: Characteristics of included trials

	Short message and protocol (n=45)	Long message (n=43)
**Number of authors randomised**	36	40
Trials per author*		
1	30 (83%)	37 (93%)
2	4 (11%)	3 (8%)
3	1 (3%)	0 (0%)
4	1 (3%)	0 (0%)
**Year of main trial publication**		
1990-2000	5 (11%)	2 (5%)
2001-2005	7 (16%)	3 (7%)
2006-2010	11 (24%)	11 (26%)
2011-2015	19 (42%)	22 (51%)
2016-2017	3 (7%)	5 (12%)
**Patients randomised**		
Mean (sd)	3910.8 [5593.4]	3755.7 [5154.8]
Median [25th, 75th centile]	1224 [439, 5170]	1809 [500, 4576]
Min, Max	74, 20332	48, 21105
**Type of trial**		
Primary prevention	20 (44%)	16 (37%)
Secondary prevention	19 (42%)	21 (49%)
Acute stroke	6 (13%)	6 (14%)
**Setting**		
1 continent	30 (67%)	17 (40%)
>1 continent	14 (31%)	23 (53%)
Not found	1 (2%)	3 (7%)
**No. of centres**		
Mean (sd)	212.8 [286.7]	165.5 [293.4]
Median [25th, 75th centile]	67 [27, 260]	85 [32, 141]
Min, Max	1, 1034	4, 1393
**Study design**		
Parallel**	44 (98%)	41 (95%)
Factorial	1 (2%)	2 (5%)
**Intervention**		
Drug	30 (67%)	31 (72%)
Surgery/procedure	12 (27%)	11 (26%)
Device	2 (4%)	1 (2%)
Diet	1 (2%)	0 (0%)
**Comparator**		
Placebo	6 (13%)	10 (23%)
Standard care	28 (62%)	24 (56%)
Active treatment	8 (18%)	6 (14%)
Surgery/procedure	3 (7%)	3 (7%)
**Number of intervention groups**		

For peer review only - http://bmjopen.bmj.com/site/about/guidelines.xhtml

2	39 (87%)	38 (88%)
3	4 (9%)	3 (7%)
4	1 (2%)	1 (2%)
>4	1 (2%)	1 (2%)
**Primary contact**		
First author	32 (71%)	30 (70%)
Second author	2 (4%)	3 (7%)
Last author	4 (9%)	5 (12%)
Other	7 (16%)	5 (12%)
Corresponding author	32 (71%)	37 (86%)
**Secondary contact**		
First author	5 (11%)	7 (16%)
Second author	8 (18%)	9 (21%)
Last author	18 (40%)	17 (40%)
Other	14 (31%)	10 (23%)
Corresponding author	4 (9%)	3 (7%)

Data are n(%) unless otherwise specified.

*Unit of allocation was carried out at author level, therefore number of trials per author do not sum to total N

**Two parallel trials were cluster randomised (Short = 1; Long = 1)

For peer review only

For peer review only - http://bmjopen.bmj.com/site/about/guidelines.xhtml

Table 2. Primary outcome: response from contacted authors

	Response	Adjusted Odds Ratio	95% C.I	p-value
**Short message and protocol**	36/45 (80%)	1.10	(0.36, 3.33)	0.87
**Long message**	33/43 (77%)			

Adjusted for year of publication, size of trial and if the author had multiple trials included. Total N included = 88. Robust standard errors were used in model fitting.

For peer review only

For peer review only - http://bmjopen.bmj.com/site/about/guidelines.xhtml

Table 3: Time to response (days)

	Time to Response				
	**Mean [sd]**	**Median [25th, 75th centile]**	**Adjusted Hazard Ratio**	**95% C.I**	**p-value**
**Short message and protocol**	51.6 [64.7]	28 [1, 77]	0.91	(0.55, 1.51)	0.72
**Long message**	60 [65.3]	42 [1, 77]			

Adjusted for year of publication, size of trial and if the author had multiple trials included. Total N included = 88.

For peer review only

For peer review only - http://bmjopen.bmj.com/site/about/guidelines.xhtml

Table 4: Secondary outcomes

	Short message and protocol (n=36)	Long message (n=40)
**Number of emails before response received**		
1	16 (44%)	15 (38%)
2	6 (17%)	10 (25%)
3	3 (8%)	3 (8%)
4	2 (6%)	2 (5%)
No response	9 (25%)	10 (25%)
Mean [sd]	1.7 [1.0]	1.7 [0.9]
Median [25th, 75th centile]	1 [1, 2]	1.5 [1, 2]
Min, Max	1, 4	1, 4
**First response outcome**		
Negative	2 (6%)	8 (20%)
Neutral	9 (25%)	14 (35%)
Positive	16 (44%)	8 (20%)
**Eventual outcome**		
Agree to collaborate	11 (31%)	10 (25%)
Do not agree to collaborate	3 (8%)	11 (28%)
No decision reached	13 (36%)	9 (23%)
No response	9 (25%)	10 (25%)

Data are n(%) unless otherwise specified. Data are presented on author level, rather than trial level.

For peer review only

For peer review only - http://bmjopen.bmj.com/site/about/guidelines.xhtml

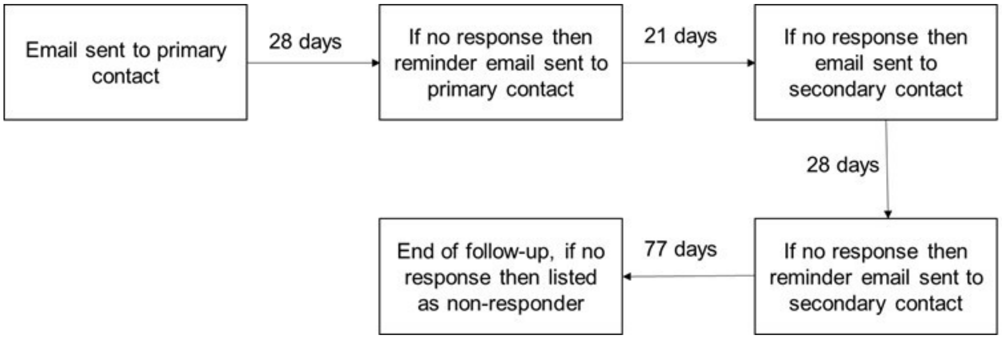

Figure 1: Plan to elicit response

159x52mm (120 x 120 DPI)

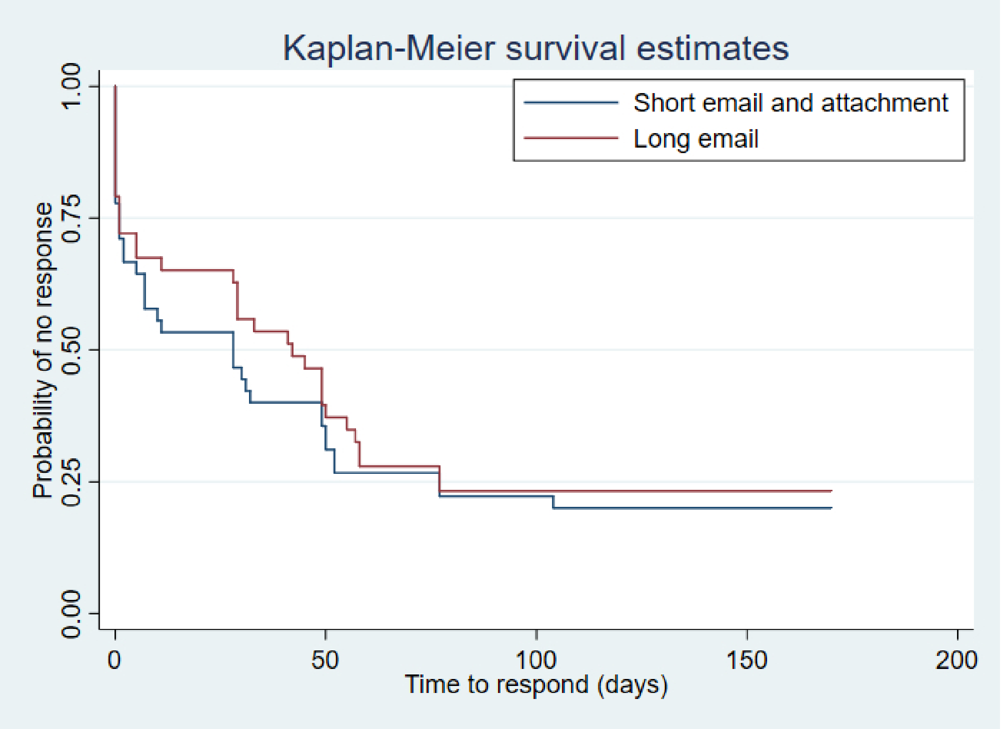

Figure 2: Kaplan-Meier curve for time to response for both intervention groups

184x134mm (96 x 96 DPI)

Appendix A:

Table A1: Sensitivity analysis. Logistic regression model for primary outcome adjusted for variables with baseline imbalance

	Response	Adjusted Odds Ratio	95% C.I	p-value
**Short message and protocol**	36/45 (80%)	0.99	(0.33, 3.03)	0.99
**Long message**	33/43 (77%)			

Adjusted for year of publication, size of trial, if the author had multiple trials included and whether the primary contact was the corresponding author. Total N included = 88. Robust standard errors were used in model fitting.

For peer review only

Appendix B: Short Invitation email

Dear (ENTER NAME HERE),

I am writing to invite you to join an international collaboration investigating the usefulness of adjudicating outcomes in stroke trials. We would like to include your trial (ENTER TRIAL NAME) in a systematic review. The collaboration is led by myself, Professor Philip Bath and Professor Alan Montgomery, all at the University of Nottingham.

It is currently unclear in what circumstances adjudication is required to produce unbiased estimates of effect. In this project, entitled Adjudicating Outcomes in Stroke Trials (AOST), we wish to compare and combine analyses of the primary outcome in trials using adjudicated and unadjudicated data.

We hope that you will be interested and willing to collaborate in this project. The AOST protocol is attached for further information. We look forward to hearing from you and thank you for taking the time to read this invitation.

Best wishes,

Peter Godolphin

NIHR Doctoral Research Fellow

For peer review only

Appendix C: Long Invitation email

Dear (ENTER NAME HERE),

I am writing to invite you to join an international collaboration investigating the usefulness of adjudicating outcomes in stroke trials. We would like to include your trial (ENTER TRIAL NAME) in a systematic review. The collaboration is led by myself, Professor Philip Bath and Professor Alan Montgomery, all at the University of Nottingham.

As you are aware, a range of outcomes are commonly adjudicated in stroke trials. It is currently unclear in what circumstances adjudication is required to produce unbiased estimates of effect. In this project, entitled Adjudicating Outcomes in Stroke Trials (AOST), we aim to address this question by combining information about adjudication from a number of stroke trials.

We wish to compare analyses of the primary outcome in trials using adjudicated and unadjudicated data. Therefore to collaborate in this project, we would like you to provide one of two things, either:

(1) Summary results from your trial (outcome in each arm, between group measure of effect and measure of variance/confidence interval) for the primary outcome based on: (i) adjudicated data, and (ii) unadjudicated data

(2) Anonymised individual participant data for key variables from your study to allow us to carry out analysis of the primary outcome using adjudicated and unadjudicated data. For this we will need:
   - Adjudicated primary outcome
   - Unadjudicated primary outcome
   - Treatment arm, covariates or other data used in primary analysis
   - Statistical analysis plan or other details of primary analysis

Data, and any accompanying materials, can be shared in any spreadsheet format (Excel, Stata, SAS etc.) and we will re-format the data to ensure that it fits our database. Data can be shared using your preferred method, for example secure download, email or CD/memory stick.

We aim to publish the results in a high impact peer-reviewed journal. All collaborators will see the final results and manuscript for comment/interpretation, and the results will be published under the banner

For peer review only - http://bmjopen.bmj.com/site/about/guidelines.xhtml

of AOST with collaborators listed by name. Our group at Nottingham has considerable experience in working with individual participant data from large, international randomised trials (including projects such as BASC, DISC, NEMAS, OAST, OT, OA-COG), and of protecting the interests of the collaborators who have shared their data/results. Your data/results will not be used for any purpose other than relating to AOST.

We believe that this study will address the lack of evidence about when adjudication of outcomes may or may not be needed in stroke trials to ensure robust results. We hope that you will be interested and willing to collaborate by sharing your data or results and (should you wish to) helping in interpretation of the results and their publication. Please let me know if you would like a copy of the AOST protocol.

We look forward to hearing from you and thank you for taking the time to read this invitation.

Best wishes,

Peter Godolphin

NIHR Doctoral Research Fellow

For peer review only

For peer review only - http://bmjopen.bmj.com/site/about/guidelines.xhtml

CONSORT 2010 checklist of information to include when reporting a randomised trial*

Section/Topic	Item No	Checklist item	Reported on page No
**Title and abstract**			
	1a	Identification as a randomised trial in the title	1
	1b	Structured summary of trial design, methods, results, and conclusions (for specific guidance see CONSORT for abstracts)	2
**Introduction**			
Background and objectives	2a	Scientific background and explanation of rationale	4/5
	2b	Specific objectives or hypotheses	5
**Methods**			
Trial design	3a	Description of trial design (such as parallel, factorial) including allocation ratio	6
	3b	Important changes to methods after trial commencement (such as eligibility criteria), with reasons	NA
Participants	4a	Eligibility criteria for participants	5
	4b	Settings and locations where the data were collected	5
Interventions	5	The interventions for each group with sufficient details to allow replication, including how and when they were actually administered	6/7 + Appendix
Outcomes	6a	Completely defined pre-specified primary and secondary outcome measures, including how and when they were assessed	7
	6b	Any changes to trial outcomes after the trial commenced, with reasons	NA
Sample size	7a	How sample size was determined	8
	7b	When applicable, explanation of any interim analyses and stopping guidelines	NA
Randomisation:			
Sequence generation	8a	Method used to generate the random allocation sequence	6
	8b	Type of randomisation; details of any restriction (such as blocking and block size)	6
Allocation concealment mechanism	9	Mechanism used to implement the random allocation sequence (such as sequentially numbered containers), describing any steps taken to conceal the sequence until interventions were assigned	6
Implementation	10	Who generated the random allocation sequence, who enrolled participants, and who assigned participants to interventions	6
Blinding	11a	If done, who was blinded after assignment to interventions (for example, participants, care providers, those	6

For peer review only - http://bmjopen.bmj.com/site/about/guidelines.xhtml

		assessing outcomes) and how	
	11b	If relevant, description of the similarity of interventions	NA
Statistical methods	12a	Statistical methods used to compare groups for primary and secondary outcomes	8
	12b	Methods for additional analyses, such as subgroup analyses and adjusted analyses	8
**Results**			
Participant flow (a diagram is strongly recommended)	13a	For each group, the numbers of participants who were randomly assigned, received intended treatment, and were analysed for the primary outcome	8
	13b	For each group, losses and exclusions after randomisation, together with reasons	NA
Recruitment	14a	Dates defining the periods of recruitment and follow-up	7/8
	14b	Why the trial ended or was stopped	NA
Baseline data	15	A table showing baseline demographic and clinical characteristics for each group	14/15
Numbers analysed	16	For each group, number of participants (denominator) included in each analysis and whether the analysis was by original assigned groups	16-19
Outcomes and estimation	17a	For each primary and secondary outcome, results for each group, and the estimated effect size and its precision (such as 95% confidence interval)	16/17
	17b	For binary outcomes, presentation of both absolute and relative effect sizes is recommended	NA
Ancillary analyses	18	Results of any other analyses performed, including subgroup analyses and adjusted analyses, distinguishing pre-specified from exploratory	19
Harms	19	All important harms or unintended effects in each group (for specific guidance see CONSORT for harms)	NA
**Discussion**			
Limitations	20	Trial limitations, addressing sources of potential bias, imprecision, and, if relevant, multiplicity of analyses	10/11
Generalisability	21	Generalisability (external validity, applicability) of the trial findings	11
Interpretation	22	Interpretation consistent with results, balancing benefits and harms, and considering other relevant evidence	9-11
**Other information**			
Registration	23	Registration number and name of trial registry	NA
Protocol	24	Where the full trial protocol can be accessed, if available	NA
Funding	25	Sources of funding and other support (such as supply of drugs), role of funders	NA

*We strongly recommend reading this statement in conjunction with the CONSORT 2010 Explanation and Elaboration for important clarifications on all the items. If relevant, we also recommend reading CONSORT extensions for cluster randomised trials, non-inferiority and equivalence trials, non-pharmacological treatments, herbal interventions, and pragmatic trials. Additional extensions are forthcoming: for those and for up to date references relevant to this checklist, see www.consort-statement.org.

For peer review only

For peer review only - http://bmjopen.bmj.com/site/about/guidelines.xhtml

BMJ Open

Short email with attachment versus long email without attachment when contacting authors to request unpublished data for a systematic review: a nested randomised trial

Journal:	*BMJ Open*
Manuscript ID	bmjopen-2018-025273.R1
Article Type:	Research
Date Submitted by the Author:	02-Oct-2018
Complete List of Authors:	Godolphin, Peter; The University of Nottingham, Nottingham Clinical Trials Unit; The University of Nottingham, Stroke Trials Unit, Division of Clinical Neuroscience<br>Bath, Philip; The University of Nottingham<br>Montgomery, Alan; University of Nottingham,
**Primary Subject Heading**:	Research methods
Secondary Subject Heading:	Research methods
Keywords:	data sharing, Stroke < NEUROLOGY, systematic review, randomised trial

SCHOLARONE™
Manuscripts

Short email with attachment versus long email without attachment when contacting authors to request unpublished data for a systematic review: a nested randomised trial

Peter J Godolphin[1,2], Philip M Bath[2], Alan A Montgomery[1]

Corresponding Author: Peter J Godolphin

Corresponding Author's Email: peter.godolphin@nottingham.ac.uk

Corresponding Author's Phone Number: +44 115 823 1563

Corresponding Author's Address:

Nottingham Clinical Trials Unit, Nottingham Health Science Partners, C Floor, South Block, Queen's Medical Centre, Derby Road, Nottingham, UK, NG7 2UH

Institutions:

1: Nottingham Clinical Trials Unit, University of Nottingham, Nottingham, UK

2: Stroke Trials Unit, Division of Clinical Neuroscience, University of Nottingham, Nottingham, UK

For peer review only - http://bmjopen.bmj.com/site/about/guidelines.xhtml

Abstract:

Objective: Systematic reviews often rely on the acquisition of unpublished analyses or data. We carried out a nested randomised trial comparing two different approaches for contacting authors to request additional data for a systematic review.

Participants: Participants were authors of published reports of prevention or treatment trials in stroke in which there was central adjudication of events. A primary and secondary research active author were selected as contacts for each trial.

Interventions: Authors were randomised to be sent either a short email with a protocol of the systematic review attached ("Short"), or a longer email that contained detailed information and without the protocol attached ("Long"). A maximum of two emails were sent to each author to obtain a response. The unit of analysis was trial, accounting for clustering by author.

Primary and secondary outcome measures: The primary outcome was whether a response was received from authors. Secondary outcomes included time to response, number of reminders needed before a response was received and whether authors agreed to collaborate.

Results: 88 trials with 76 primary authors were identified in the systematic review, and of these, 36 authors were randomised to Short [trials=45], and 40 to Long [trials=43]. Responses were received for 69 trials. There was no evidence of a difference in response rate between trial arms (Short vs Long, odds ratio 1.10, 95% C.I: [0.36, 3.33]). There was no evidence of a difference in time to response between trial arms (Short vs Long, hazard ratio 0.91, 95% C.I: [0.55, 1.51]). In total, 27% of authors responded within a day and 22% of authors never responded.

Conclusions: There was no evidence to suggest that email format had an impact on the number of responses received when acquiring data for a systematic review involving stroke trials, or the time taken to receive these responses.

Keywords: data sharing, clinical trial, stroke, systematic review

For peer review only - http://bmjopen.bmj.com/site/about/guidelines.xhtml

Article summary:

Strength and limitations of this study:

- This is the first randomised trial comparing different email formats when contacting authors to request unpublished data for a systematic review.
- This study follows a clear and rigorous protocol, guided by experienced methodologists, and implemented in a clinical trials unit.
- Blinding was not possible, as it was clear which format of email had been sent. However contacted authors were not aware of this trial during the study and therefore we expected that their response rate and time to response would remain unbiased.
- The sample size is constrained by the number of eligible studies identified for the systematic review. It is possible that a larger study would detect small but important effects of email format and presence or absence of an attachment on response rate.

For peer review only

Introduction:

Systematic reviews are often considered as the highest level of evidence available[1]. However, meta-analyses, the statistical component of a systematic review, often rely on the acquisition of unpublished summary results or further data. It can be a challenge to obtain this data, especially if the meta-analysis requires individual patient data[2, 3], resulting in insufficient studies to pool together[4].

Data sharing is not yet common practice in the field of biomedical research and many researchers struggle to acquire the underlying datasets used in journal articles[5]. There are many reasons why there may be difficulties in acquiring data; authors may not wish to share their data[6], they may be too busy to respond or deal with requests[4], they may have moved institution and be uncontactable, the data could have been lost or destroyed, or authors may not have access to the data[7]. Author inclination to share data could be related to the strength of evidence and the perceived quality of statistical results from their study[8]. Research studies that report borderline evidence of an intervention effect may be less likely to take part in data sharing, which would result in publication bias for a systematic review[9]. Various methods are currently employed to encourage authors to share data for a systematic review, and a randomised trial investigating if financial incentives may be effective is currently underway[10]. Additionally, academic incentives for making data available could be introduced, such as invitation into group collaboration, or having data sharing recognised to an equivalent level as scientific presentations or journal publications[11].

There appears to be no consensus about the best way to approach authors to contact them for data. The corresponding author should be the most appropriate person to contact when attempting to acquire unpublished clinical trial data, but contacting these authors may be challenging as they could be inaccessible or have little time to deal with requests[12]. Therefore, if email contact is used, this must be clear, concise and easy to respond to. Some authors may prefer a shorter email, with additional information attached, whilst others may prefer a longer email with sufficient information they need to make a decision without reading additional attached information such as a study protocol.

The initial interaction with any author may have a large impact on whether they collaborate, or even on whether they reply at all. The first step when attempting to acquire data is to open up a channel of communication with the data custodian, assumed in this paper to be the author of the journal article.

For peer review only - http://bmjopen.bmj.com/site/about/guidelines.xhtml

Thus, an important outcome to assess is whether an initial response is received, before considering if interaction with the author affects willingness to share data.

We carried out a nested randomised trial, which investigated two different approaches to contacting authors for data in a systematic review, with the primary aim of seeing which method elicits the most complete response.

Methods:

Participants:

The systematic review chosen for this study investigates whether central adjudication of the primary outcome in stroke trials has any impact on the main trial primary analysis[13]. Since the information required for the review is not commonly published in the main or secondary trial publications, author contact is essential which makes it a useful setting to test the effect of different ways of contacting authors on response rate.

In brief, the studies included were those adjudged to be randomised trials of prevention or treatment of stroke, that had centrally adjudicated their primary outcome. MEDLINE, Embase, the Cochrane Central Register of Controlled Trials, Web of Science, PsycINFO and Google Scholar were searched for relevant articles. We restricted searches from Google Scholar to the first 300 articles[14], and only articles written in English were considered.

Two author contacts were chosen from each identified trial and these were the participants selected for this study. The primary contact was ideally one of the corresponding authors. This author was checked on PubMed to identify whether they were still an active researcher (paper published within the last 2 years). If the corresponding author appeared to have ceased research activity, then a secondary author who was still an active researcher (preferably first/second/last) was selected as the primary point of contact. Additionally, a second research active author was chosen as a second contact for the trial. Ideally, this second contact also had a major role in the trial, determined by their position on the main trial publication and in the author contribution section of the manuscript, if completed.

For peer review only - http://bmjopen.bmj.com/site/about/guidelines.xhtml

Authors were invited to collaborate in our research programme investigating adjudication in stroke trials. In order to collaborate, they were asked to provide data in one of two formats: individual participant data or summary results. Authors that provided data were given the opportunity to critically review the findings and draft manuscript. Those that accepted this offer were invited to join the systematic review writing committee.

Patient and Public Involvement:

Patients were not involved in this study.

Trial Design:

This study was a parallel group, randomised trial of short email and attachment versus long email to elicit response from potential collaborators. The trial was nested within a systematic review and meta-analysis. Blinding was not possible, as it was clear to contacted authors and the trial team which format of email had been sent. However, contacted authors were unaware that alternative formats of contact email were being compared in a nested randomised study and therefore we expected that their willingness to respond would be unbiased.

Randomisation:

Randomisation occurred at author level. This was to ensure that if any author had published multiple trials that were to be included, then they only received one email, rather than multiple emails, which could be in different formats. Randomisation was stratified by year of publication (median split: <2011/≥2011), size of trial (median split: <1738/≥1738) and if the author had multiple trials which are included (yes/no). Therefore, there were eight strata. If authors had multiple trials, then the within-author median size and median year were used when stratifying. Allocation was concealed and a researcher not involved in the trial randomised participants using a computer-generated random number list.

Participants were randomised in a 1:1 ratio to either of the two treatment groups: (1) Short, where they received a short email message that fits on one screen without the need to scroll, with the protocol attached (Intervention group); (2) Long, where they received a longer email message that contained a fuller description of the systematic review and request for information, with the protocol

For peer review only - http://bmjopen.bmj.com/site/about/guidelines.xhtml

available on request (Comparator group). The protocol detailed the purpose of the systematic review, the data that was requested and the planned programme of research (see Appendices *A* & *B*).

Plan for eliciting response:

Each author received a maximum of two emails. The primary contact was first sent an email in the format of the randomly allocated intervention. If no response was received, then a second email was sent 28 days later, which included some additional brief reminder text, which was the same for both groups, added at the top of the original email (achieved using Reply to all in Outlook).

If there was still no response from the primary contact author after a further 21 days, the second contact author was sent an email in an identical format, waiting 28 days before a reminder was sent. The second contact was not emailed if the first contact responded within the seven-week timeframe. Therefore, there was a maximum of four emails in total that authors received if there was no response. Participants were followed up for a further eleven weeks after the fourth and final email. After this stage, any authors that had not responded were recorded as non-respondents, regardless of contact thereafter (Figure 1).

Outcome Measures:

The primary outcome for this nested randomised trial was whether a response was received from either of the two contacted authors in the trial timeframe. This was a binary outcome (Response received, Yes/No). Out of office automatic replies were not counted as a response, and no additional emails were sent upon receipt of an out of office reply.

Secondary outcomes were time to response, measured in days, and the number of emails needed to respond. Upon receipt of an initial response, this was categorised as either being Negative, Neutral or Positive. Positive responses were allocated if there was an interest in the collaboration and research questions, above simply asking for further information. Negative responses were assigned if participants gave the indication that they did not want to be involved/did not have time to take part. All other responses were coded as Neutral.

The eventual outcome upon the end of the trial timeframe was allocated as either (1) agree to collaborate, (2) do not agree to collaborate, (3) no decision reached or (4) no response. If there was an agreement in principle regardless of whether data had been received, then this was assigned as

For peer review only - http://bmjopen.bmj.com/site/about/guidelines.xhtml

For peer review only

agree. If there was a clear indication that the author could not/did not want to help then this was allocated do not agree. No decision reached was given if it was still unclear whether the author would be willing to take part in the collaboration.

Statistical Analysis:

The sample size for this nested randomised trial was fixed by the number of studies identified in the systematic review, and although allocation was by author, the unit of analysis is the individual trial. A sample size of 88 trials could detect a difference in response rate between groups of ≤30 percentage points (equivalent odds ratio 3.4) with 80% power and 5% two-sided alpha, ignoring any reduction in effective sample size due to the clustering effect of authors with multiple studies. The characteristics of each trial were summarised using appropriate descriptive statistics for each intervention arm. The primary approach to between-group comparative analyses was by intention-to-treat. Short was treated as the intervention group and Long treated as the comparator group for all between group analyses. The evaluation of response rate was performed using adjusted logistic regression models. The adjusted model included stratification factors; year of trial publication, trial sample size and multiple trials per author. The primary efficacy parameter comparing interventions was the odds ratio along with the corresponding 95% confidence interval. Robust standard errors were used to account for correlation between multiple trials for the same author. A sensitivity analysis was carried out by further adjusting the primary analysis model for any characteristics of trials with marked imbalance between intervention groups.

Time to response was investigated using survival analysis, with response being the outcome of interest. Adjusted hazard ratios, adjusted for stratification factors, were reported alongside 95% confidence intervals. Additional secondary outcomes were summarised using mean, standard deviation, median, lower and upper quartiles, or frequency counts and percentages where appropriate. All analyses were performed in Stata version 15.0 or later.

Results:

The study commenced on 11th July 2017. 88 trials were identified from a systematic search of the literature and were to be included in the review. This corresponded to 76 unique authors who were

For peer review only - http://bmjopen.bmj.com/site/about/guidelines.xhtml

selected as the primary contact for these studies. 36 authors were randomised to Short (45 trials), and 40 to Long (43 trials) email formats. The majority of the studies had their main publication published between 2006 and 2015, were either primary or secondary prevention stroke trials, were carried out in over 50 trial centres and had a median number of patients randomised above 1000 (Table 1). Trials randomised to each group had similar study design, intervention type and comparator, but studies allocated to Long tended to have more participants included and a larger number of trial centres. The number of intervention groups was similar for studies allocated to either Short or Long.

The majority of authors chosen as the primary contact were the first author of the main publication, or listed first in the writing committee if the article was published under a collaborative group. Conversely, the last author was the most common choice for the second contact, although there was greater variation in choice for this contact, with a position other than first, second or last also prevalent. For studies allocated to Long, the primary contact was more likely to be the corresponding author compared to trials allocated to Short, although author position was similar for both groups (Table 1).

In all, responses were received for 69 trials, with authors of 19 studies (22%) never responding. There was no evidence of a difference in response rate between trial arms (Table 2 & *Appendix C*). Average time to response was marginally quicker among those allocated to Short, with a greater proportion of authors in this trial arm responding to the first email, but there was insufficient evidence to determine a difference between trial arms on time to response (Table 3 & Figure 2). In total, 27% of authors responded within a day and 38% replied within a week.

A short email elicited a more favourable first response, with few negative replies. However, this did not lead to a larger number of authors agreeing to collaborate, with many authors not following up on their positive intent. Conversely, those allocated the longer email were more likely to respond in a negative fashion, but this meant that the decision not to collaborate was established sooner, and there was a smaller proportion with no decision reached (Table 4).

Discussion:

For peer review only - http://bmjopen.bmj.com/site/about/guidelines.xhtml

This nested randomised trial found no evidence that email format or presence or absence of an attachment had an impact on author response when attempting to acquire data for a systematic review. Authors responded in a similar timeframe, and needed a similar number of reminders before a response was received. There was some indication that authors that received a shorter email were more likely to respond positively at first, but there was no evidence that email format changed the likelihood of collaboration.

Approximately three-quarters of authors in the study responded regardless of email format. A study which attempted to contact authors to obtain data for a diagnostic accuracy review for hepatic fibrosis found a similar response rate, with 68% of authors responding to requests for data[15]. For both email formats we included text which invited authors who provided data into a collaboration to assist (at their discretion) with interpretation and write-up of results, and co-authorship as part of a collaborative group. Our view was that offering greater opportunity for collaboration to authors rather than simply providing data or further results might produce a greater and more complete response. Offering collaboration may have influenced overall response rate, and thus the results of this study may not be generalisable to others where collaboration is not offered. For example, a study which aimed to establish whether corresponding authors accepted responsibility of correspondence, where collaboration was not offered, found a far lower response rate (190/446, 43%)[12]. A qualitative interview study which investigated strategies to access unpublished clinical trial data mentioned a lack of collaboration as a main barrier to data sharing[16]. Thus, offering the opportunity for authors to collaborate and being upfront about giving collaborators the chance to input in both interpreting results and the write-up of any papers could give a more positive and thorough response.

Whilst short emails elicited a marginally quicker response on average, there was insufficient evidence to conclude benefit of this email style on time to response. Selph et al.[15] found that responses were received from 23% of authors after one request, with 55% responding after just two requests (one reminder). This is similar to our study, where we found that 41% of authors had responded after one email and two emails (one reminder) elicited 62% response rate. A third email provided 70% response rate, with little further improvement following a fourth email. The number of emails sent and the number sent to each contact (if there are multiple like in our study) are additional areas where similar research could help to introduce a more rigorous evidence base.

For peer review only - http://bmjopen.bmj.com/site/about/guidelines.xhtml

There was some indication that authors allocated to a short email format were more likely to respond positively, with only two negative first responses received from these authors, and double the number of positive responses. However, the number of authors eventually agreeing to collaborate was similar for both groups. This suggests that whilst the long email format resulted in more negative initial responses, authors who did respond were aware of the implications of our study and could make an informed decision. Thus, the majority of positive first responses to those allocated to the long email format turned into collaboration. It could be hypothesised that whilst those allocated to the short email format had a detailed protocol attached, authors may not have opened attachments and therefore were agreeing to be involved in the study without fully realised the commitment. This led to a far larger amount of time and resource dealing with these authors, who eventually declined to collaborate, or no decision was reached in the trial timeframe.

A limitation of this study was the constrained sample size, enforced by the systematic within which this study was nested. Thus, power to detect a small difference in response rate was low. However, this study does provide a framework to carry out a larger trial of this kind. A further limitation of our trial was that blinding was not possible, because the investigator was responsible for sending all emails and collating the data. It was impossible to mask which intervention participants were assigned to, due to the email thread being used to reply to messages and send reminders. Therefore, there is a possibility that some outcomes are at a risk of bias. Furthermore, the eventual outcome was not expected to be greatly influenced by the intervention, and was more reliant on subsequent communication such as emails or phone contact. However, the protocol was finalised in advance of randomisation, and the trial team followed this protocol, therefore the risk of bias from the trial team was low. In addition, study authors, although aware of email format they received, were not aware of the alternative email format nor were they aware of the nested randomised study, therefore the risk of bias from participants was low.

It could be argued that this study did not investigate what could be perceived as the key question, whether data was shared. This was due to our belief that whether authors provided data was dependent on other factors, and not the original email length and style. Our study investigated an intervention which allows researchers to open up a channel of communication with the data custodian, and there is the potential to use a different intervention to evaluate how best to acquire data (for example financial incentives[10]).

For peer review only - http://bmjopen.bmj.com/site/about/guidelines.xhtml

This nested randomised trial found no evidence that email length had an impact on author response, or agreement to collaborate, when attempting to acquire data in our systematic review. Further studies could test similar hypotheses to evaluate this further, with a greater sample size and in a different clinical area. Given this issue involves countless researchers who strive to generate high quality research through systematic reviews, this problem seems one that is not only important to tackle, but could be potentially simple to answer. Whilst the first question has to be whether an author responds, the more important question to answer is whether or not an author agrees to collaborate, and arguably even more important, whether or not data is shared. These questions go far beyond the scope of this small trial, but future studies building on this could investigate these issues and attempt to find an intervention that not only boosts response, but also boosts collaboration.

Contributors: All authors conceived the study. PJG wrote the analysis plan. AAM and PMB reviewed the analysis plan. PJG collected the data, carried out interventions and undertook data analysis. PJG, AAM, PMB interpreted the data. PJG wrote the first draft of the manuscript. AAM, PMB commented on successive versions of the manuscript for important intellectual content. All authors approved the final version of the manuscript.

Funding: PJG was funded for this summary of independent research by the National Institute for Health Research (NIHR)'s Doctoral Research Fellowship Programme (DRF-2016-09-057). The views expressed are those of the authors and not necessarily those of the NHS, the NIHR or the Department of Health. PMB is Stroke Association Professor of Stroke Medicine and is a NIHR Senior Investigator.

Competing interests: None

Ethical approval: As this was a randomised trial of a methodology rather than a treatment involving patients, we considered that prior consent and trial registration were not appropriate. Furthermore, the first contact with participants had to be randomised otherwise this would negate the purpose of this study.

For peer review only - http://bmjopen.bmj.com/site/about/guidelines.xhtml

Data sharing: The datasets generated during this study will be available upon request from Peter Godolphin (peter.godolphin@nottingham.ac.uk), a minimum of 6 months after publication. Access to the data will be subject to a review of a data sharing and use request by a committee, and will only be granted upon receipt of a data sharing and use agreement. Any data shared will be anonymised which may affect the reproducibility of published analysis.

References

1. Paul, M. and L. Leibovici, *Systematic review or meta-analysis? Their place in the evidence hierarchy.* Clinical Microbiology and Infection, 2014. **20**(2): p. 97-100.
2. Cooper, H. and E.A. Patall, *The relative benefits of meta-analysis conducted with individual participant data versus aggregated data.* Psychol Methods, 2009. **14**(2): p. 165-76.
3. Polanin, J.R., *Efforts to retrieve individual participant data sets for use in a meta-analysis result in moderate data sharing but many data sets remain missing.* Journal of Clinical Epidemiology, 2018. **98**: p. 157-159.
4. Jaspers, G.J. and P.L. Degraeuwe, *A failed attempt to conduct an individual patient data meta-analysis.* Syst Rev, 2014. **3**: p. 97.
5. Hrynaszkiewicz, I., et al., *Publishing descriptions of non-public clinical datasets: proposed guidance for researchers, repositories, editors and funding organisations.* Research Integrity and Peer Review, 2016. **1**(1): p. 6.
6. Savage, C.J. and A.J. Vickers, *Empirical Study of Data Sharing by Authors Publishing in PLoS Journals.* PLOS ONE, 2009. **4**(9): p. e7078.
7. Lundh, A., L.T. Krogsbøll, and P.C. Gøtzsche, *Access to data in industry-sponsored trials.* The Lancet. **378**(9808): p. 1995-1996.
8. Wicherts, J.M., M. Bakker, and D. Molenaar, *Willingness to Share Research Data Is Related to the Strength of the Evidence and the Quality of Reporting of Statistical Results.* PLOS ONE, 2011. **6**(11): p. e26828.
9. Ahmed, I., A.J. Sutton, and R.D. Riley, *Assessment of publication bias, selection bias, and unavailable data in meta-analyses using individual participant data: a database survey.* BMJ, 2012. **344**.
10. Veroniki, A.A., et al., *Contacting authors to retrieve individual patient data: study protocol for a randomized controlled trial.* Trials, 2016. **17**(1): p. 138.
11. Ohmann, C., et al., *Sharing and reuse of individual participant data from clinical trials: principles and recommendations.* BMJ Open, 2017. **7**(12).
12. Teunis, T., S.P.F.T. Nota, and J.H. Schwab, *Do Corresponding Authors Take Responsibility for Their Work? A Covert Survey.* Clinical Orthopaedics and Related Research®, 2015. **473**(2): p. 729-735.
13. Godolphin, P., A. Montgomery, and P. Bath. *A comparison of independent adjudication and site assessment of outcomes and its impact on treatment effect estimates in randomised stroke trials: systematic review and meta-analyses*. 2017 [cited 2018 22/03/2018]; Available from: http://www.crd.york.ac.uk/PROSPERO/display_record.php?ID=CRD42017056731.
14. Haddaway, N.R., et al., *The Role of Google Scholar in Evidence Reviews and Its Applicability to Grey Literature Searching.* PLOS ONE, 2015. **10**(9): p. e0138237.
15. Selph, S.S., A.D. Ginsburg, and R. Chou, *Impact of contacting study authors to obtain additional data for systematic reviews: diagnostic accuracy studies for hepatic fibrosis.* Systematic Reviews, 2014. **3**: p. 107-107.
16. Wolfe, N., P.C. Gøtzsche, and L. Bero, *Strategies for obtaining unpublished drug trial data: a qualitative interview study.* Systematic reviews, 2013. **2**(1): p. 31.

For peer review only - http://bmjopen.bmj.com/site/about/guidelines.xhtml

For peer review only

For peer review only - http://bmjopen.bmj.com/site/about/guidelines.xhtml

Tables:

Table 1: Characteristics of included trials

	Short message and protocol (n=45)	Long message (n=43)
**Number of authors randomised**	36	40
Trials per author*		
1	30 (83%)	37 (93%)
2	4 (11%)	3 (8%)
3	1 (3%)	0 (0%)
4	1 (3%)	0 (0%)
**Year of main trial publication**		
1990-2000	5 (11%)	2 (5%)
2001-2005	7 (16%)	3 (7%)
2006-2010	11 (24%)	11 (26%)
2011-2015	19 (42%)	22 (51%)
2016-2017	3 (7%)	5 (12%)
**Patients randomised**		
Mean (sd)	3910.8 [5593.4]	3755.7 [5154.8]
Median [25th, 75th centile]	1224 [439, 5170]	1809 [500, 4576]
Min, Max	74, 20332	48, 21105
**Type of trial**		
Primary prevention	20 (44%)	16 (37%)
Secondary prevention	19 (42%)	21 (49%)
Acute stroke	6 (13%)	6 (14%)
**Setting**		
1 continent	30 (67%)	17 (40%)
>1 continent	14 (31%)	23 (53%)
Not found	1 (2%)	3 (7%)
**No. of centres**		
Mean (sd)	212.8 [286.7]	165.5 [293.4]
Median [25th, 75th centile]	67 [27, 260]	85 [32, 141]
Min, Max	1, 1034	4, 1393
**Study design**		
Parallel**	44 (98%)	41 (95%)
Factorial	1 (2%)	2 (5%)
**Intervention**		
Drug	30 (67%)	31 (72%)
Surgery/procedure	12 (27%)	11 (26%)
Device	2 (4%)	1 (2%)
Diet	1 (2%)	0 (0%)
**Comparator**		
Placebo	6 (13%)	10 (23%)
Standard care	28 (62%)	24 (56%)
Active treatment	8 (18%)	6 (14%)
Surgery/procedure	3 (7%)	3 (7%)
**Number of intervention groups**		

For peer review only - http://bmjopen.bmj.com/site/about/guidelines.xhtml

2	39 (87%)	38 (88%)
3	4 (9%)	3 (7%)
4	1 (2%)	1 (2%)
>4	1 (2%)	1 (2%)
**Primary contact**		
First author	32 (71%)	30 (70%)
Second author	2 (4%)	3 (7%)
Last author	4 (9%)	5 (12%)
Other	7 (16%)	5 (12%)
Corresponding author	32 (71%)	37 (86%)
**Secondary contact**		
First author	5 (11%)	7 (16%)
Second author	8 (18%)	9 (21%)
Last author	18 (40%)	17 (40%)
Other	14 (31%)	10 (23%)
Corresponding author	4 (9%)	3 (7%)

Data are n(%) unless otherwise specified.
*Unit of allocation was carried out at author level, therefore number of trials per author do not sum to total N
**Two parallel trials were cluster randomised (Short = 1; Long = 1)

For peer review only - http://bmjopen.bmj.com/site/about/guidelines.xhtml

Table 2. Primary outcome: response from contacted authors

	Response	Adjusted Odds Ratio	95% C.I	p-value
**Short message and protocol**	36/45 (80%)	1.10	(0.36, 3.33)	0.87
**Long message**	33/43 (77%)			

Adjusted for year of publication, size of trial and if the author had multiple trials included. Total N included = 88. Robust standard errors were used in model fitting.

For peer review only

For peer review only - http://bmjopen.bmj.com/site/about/guidelines.xhtml

Table 3: Time to response (days)

	Time to Response				
	**Mean [sd]**	**Median [25th, 75th centile]**	**Adjusted Hazard Ratio**	**95% C.I**	**p-value**
**Short message and protocol**	51.6 [64.7]	28 [1, 77]	0.91	(0.55, 1.51)	0.72
**Long message**	60 [65.3]	42 [1, 77]			

Adjusted for year of publication, size of trial and if the author had multiple trials included. Total N included = 88.

For peer review only

For peer review only - http://bmjopen.bmj.com/site/about/guidelines.xhtml

Table 4: Secondary outcomes

	Short message and protocol (n=36)	Long message (n=40)
**Number of emails before response received**		
1	16 (44%)	15 (38%)
2	6 (17%)	10 (25%)
3	3 (8%)	3 (8%)
4	2 (6%)	2 (5%)
No response	9 (25%)	10 (25%)
Mean [sd]	1.7 [1.0]	1.7 [0.9]
Median [25th, 75th centile]	1 [1, 2]	1.5 [1, 2]
Min, Max	1, 4	1, 4
**First response outcome**		
Negative	2 (6%)	8 (20%)
Neutral	9 (25%)	14 (35%)
Positive	16 (44%)	8 (20%)
**Eventual outcome**		
Agree to collaborate	11 (31%)	10 (25%)
Do not agree to collaborate	3 (8%)	11 (28%)
No decision reached	13 (36%)	9 (23%)
No response	9 (25%)	10 (25%)

Data are n(%) unless otherwise specified. Data are presented on author level, rather than trial level.

For peer review only

For peer review only - http://bmjopen.bmj.com/site/about/guidelines.xhtml

Figure Legends:

Figure 1: Plan to elicit response

Figure 2: Kaplan-Meier curve for time to response for both intervention groups

For peer review only

For peer review only - http://bmjopen.bmj.com/site/about/guidelines.xhtml

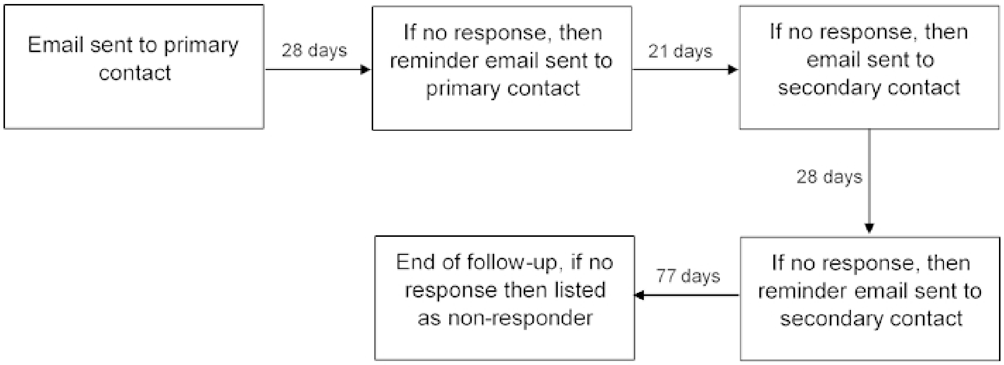

Plan to elicit response

59x21mm (300 x 300 DPI)

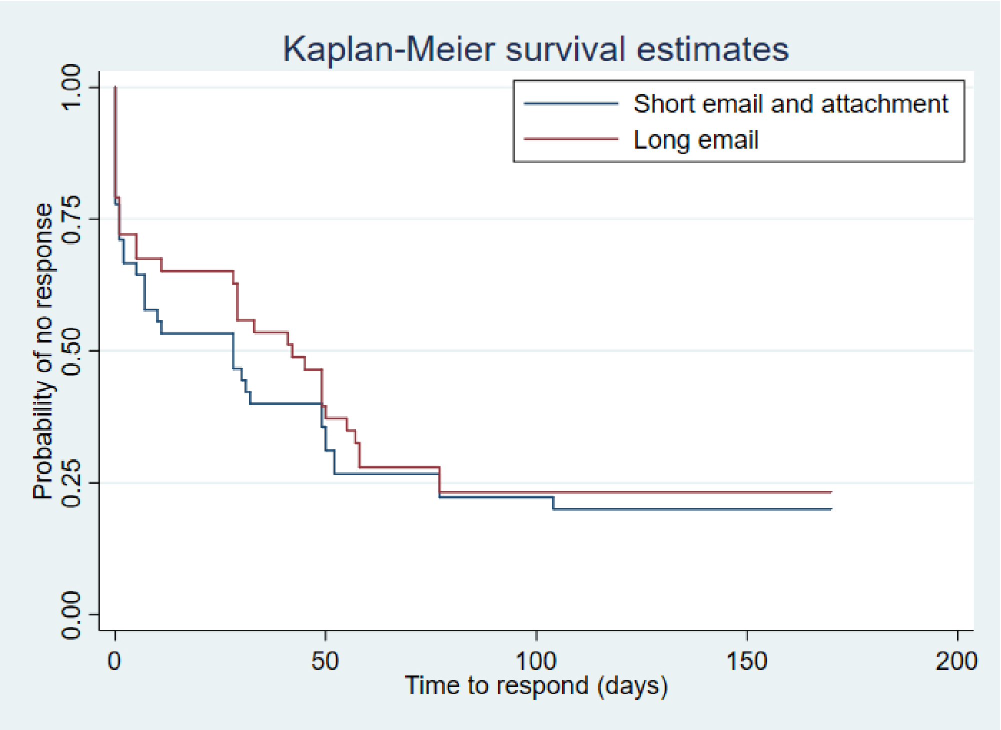

Kaplan-Meier curve for time to response for both intervention groups

59x43mm (300 x 300 DPI)

Appendix A: Short Invitation email

Dear (ENTER NAME HERE),

I am writing to invite you to join an international collaboration investigating the usefulness of adjudicating outcomes in stroke trials. We would like to include your trial (ENTER TRIAL NAME) in a systematic review. The collaboration is led by myself, Professor Philip Bath and Professor Alan Montgomery, all at the University of Nottingham.

It is currently unclear in what circumstances adjudication is required to produce unbiased estimates of effect. In this project, entitled Adjudicating Outcomes in Stroke Trials (AOST), we wish to compare and combine analyses of the primary outcome in trials using adjudicated and unadjudicated data.

We hope that you will be interested and willing to collaborate in this project. The AOST protocol is attached for further information. We look forward to hearing from you and thank you for taking the time to read this invitation.

Best wishes,

Peter Godolphin

NIHR Doctoral Research Fellow

For peer review only

Appendix B: Long Invitation email

Dear (ENTER NAME HERE),

I am writing to invite you to join an international collaboration investigating the usefulness of adjudicating outcomes in stroke trials. We would like to include your trial (ENTER TRIAL NAME) in a systematic review. The collaboration is led by myself, Professor Philip Bath and Professor Alan Montgomery, all at the University of Nottingham.

As you are aware, a range of outcomes are commonly adjudicated in stroke trials. It is currently unclear in what circumstances adjudication is required to produce unbiased estimates of effect. In this project, entitled Adjudicating Outcomes in Stroke Trials (AOST), we aim to address this question by combining information about adjudication from a number of stroke trials.

We wish to compare analyses of the primary outcome in trials using adjudicated and unadjudicated data. Therefore to collaborate in this project, we would like you to provide one of two things, either:

(1) Summary results from your trial (outcome in each arm, between group measure of effect and measure of variance/confidence interval) for the primary outcome based on: (i) adjudicated data, and (ii) unadjudicated data

(2) Anonymised individual participant data for key variables from your study to allow us to carry out analysis of the primary outcome using adjudicated and unadjudicated data. For this we will need:

- Adjudicated primary outcome
- Unadjudicated primary outcome
- Treatment arm, covariates or other data used in primary analysis
- Statistical analysis plan or other details of primary analysis

Data, and any accompanying materials, can be shared in any spreadsheet format (Excel, Stata, SAS etc.) and we will re-format the data to ensure that it fits our database. Data can be shared using your preferred method, for example secure download, email or CD/memory stick.

We aim to publish the results in a high impact peer-reviewed journal. All collaborators will see the final results and manuscript for comment/interpretation, and the results will be published under the banner

For peer review only - http://bmjopen.bmj.com/site/about/guidelines.xhtml

of AOST with collaborators listed by name. Our group at Nottingham has considerable experience in working with individual participant data from large, international randomised trials (including projects such as BASC, DISC, NEMAS, OAST, OT, OA-COG), and of protecting the interests of the collaborators who have shared their data/results. Your data/results will not be used for any purpose other than relating to AOST.

We believe that this study will address the lack of evidence about when adjudication of outcomes may or may not be needed in stroke trials to ensure robust results. We hope that you will be interested and willing to collaborate by sharing your data or results and (should you wish to) helping in interpretation of the results and their publication. Please let me know if you would like a copy of the AOST protocol.

We look forward to hearing from you and thank you for taking the time to read this invitation.

Best wishes,

Peter Godolphin

NIHR Doctoral Research Fellow

For peer review only - http://bmjopen.bmj.com/site/about/guidelines.xhtml

Appendix C:

Table A1: Sensitivity analysis. Logistic regression model for primary outcome adjusted for variables with baseline imbalance

	Response	Adjusted Odds Ratio	95% C.I	p-value
**Short message and protocol**	36/45 (80%)	0.99	(0.33, 3.03)	0.99
**Long message**	33/43 (77%)			

Adjusted for year of publication, size of trial, if the author had multiple trials included and whether the primary contact was the corresponding author. Total N included = 88. Robust standard errors were used in model fitting.

For peer review only

CONSORT 2010 checklist of information to include when reporting a randomised trial*

Section/Topic	Item No	Checklist item	Reported on page No
**Title and abstract**			
	1a	Identification as a randomised trial in the title	1
	1b	Structured summary of trial design, methods, results, and conclusions (for specific guidance see CONSORT for abstracts)	2
**Introduction**			
Background and objectives	2a	Scientific background and explanation of rationale	4/5
	2b	Specific objectives or hypotheses	5
**Methods**			
Trial design	3a	Description of trial design (such as parallel, factorial) including allocation ratio	6
	3b	Important changes to methods after trial commencement (such as eligibility criteria), with reasons	NA
Participants	4a	Eligibility criteria for participants	5
	4b	Settings and locations where the data were collected	5
Interventions	5	The interventions for each group with sufficient details to allow replication, including how and when they were actually administered	6/7 + Appendix
Outcomes	6a	Completely defined pre-specified primary and secondary outcome measures, including how and when they were assessed	7
	6b	Any changes to trial outcomes after the trial commenced, with reasons	NA
Sample size	7a	How sample size was determined	8
	7b	When applicable, explanation of any interim analyses and stopping guidelines	NA
Randomisation:			
Sequence generation	8a	Method used to generate the random allocation sequence	6
	8b	Type of randomisation; details of any restriction (such as blocking and block size)	6
Allocation concealment mechanism	9	Mechanism used to implement the random allocation sequence (such as sequentially numbered containers), describing any steps taken to conceal the sequence until interventions were assigned	6
Implementation	10	Who generated the random allocation sequence, who enrolled participants, and who assigned participants to interventions	6
Blinding	11a	If done, who was blinded after assignment to interventions (for example, participants, care providers, those	6

For peer review only

For peer review only - http://bmjopen.bmj.com/site/about/guidelines.xhtml

		assessing outcomes) and how	
	11b	If relevant, description of the similarity of interventions	NA
Statistical methods	12a	Statistical methods used to compare groups for primary and secondary outcomes	8
	12b	Methods for additional analyses, such as subgroup analyses and adjusted analyses	8
**Results**			
Participant flow (a diagram is strongly recommended)	13a	For each group, the numbers of participants who were randomly assigned, received intended treatment, and were analysed for the primary outcome	8
	13b	For each group, losses and exclusions after randomisation, together with reasons	NA
Recruitment	14a	Dates defining the periods of recruitment and follow-up	7/8
	14b	Why the trial ended or was stopped	NA
Baseline data	15	A table showing baseline demographic and clinical characteristics for each group	14/15
Numbers analysed	16	For each group, number of participants (denominator) included in each analysis and whether the analysis was by original assigned groups	16-19
Outcomes and estimation	17a	For each primary and secondary outcome, results for each group, and the estimated effect size and its precision (such as 95% confidence interval)	16/17
	17b	For binary outcomes, presentation of both absolute and relative effect sizes is recommended	NA
Ancillary analyses	18	Results of any other analyses performed, including subgroup analyses and adjusted analyses, distinguishing pre-specified from exploratory	19
Harms	19	All important harms or unintended effects in each group (for specific guidance see CONSORT for harms)	NA
**Discussion**			
Limitations	20	Trial limitations, addressing sources of potential bias, imprecision, and, if relevant, multiplicity of analyses	10/11
Generalisability	21	Generalisability (external validity, applicability) of the trial findings	11
Interpretation	22	Interpretation consistent with results, balancing benefits and harms, and considering other relevant evidence	9-11
**Other information**			
Registration	23	Registration number and name of trial registry	NA
Protocol	24	Where the full trial protocol can be accessed, if available	NA
Funding	25	Sources of funding and other support (such as supply of drugs), role of funders	NA

*We strongly recommend reading this statement in conjunction with the CONSORT 2010 Explanation and Elaboration for important clarifications on all the items. If relevant, we also recommend reading CONSORT extensions for cluster randomised trials, non-inferiority and equivalence trials, non-pharmacological treatments, herbal interventions, and pragmatic trials. Additional extensions are forthcoming: for those and for up to date references relevant to this checklist, see www.consort-statement.org.

For peer review only

For peer review only - http://bmjopen.bmj.com/site/about/guidelines.xhtml
